# 14-3-3τ as a Modulator of Early α-Synuclein Multimerization and Amyloid Formation

**DOI:** 10.1021/acschemneuro.4c00100

**Published:** 2024-04-18

**Authors:** Gobert Heesink, Maxime C. M. van den Oetelaar, Slav A. Semerdzhiev, Christian Ottmann, Luc Brunsveld, Christian Blum, Mireille M. A. E. Claessens

**Affiliations:** † Nanobiophysics, Faculty of Science and Technology, MESA + Institute for Nanotechnology and Technical Medical Centre, University of Twente, Enschede 7500 AE, The Netherlands; ‡ Laboratory of Chemical Biology, Department of Biomedical Engineering and Institute for Complex Molecular Systems, Eindhoven University of Technology, Eindhoven 5600 MB, The Netherlands

**Keywords:** α-synuclein aggregation, 14-3-3 chaperone, IDP multimerization, modulation of multimerization, protein co-condensation, protein-protein interactions

## Abstract

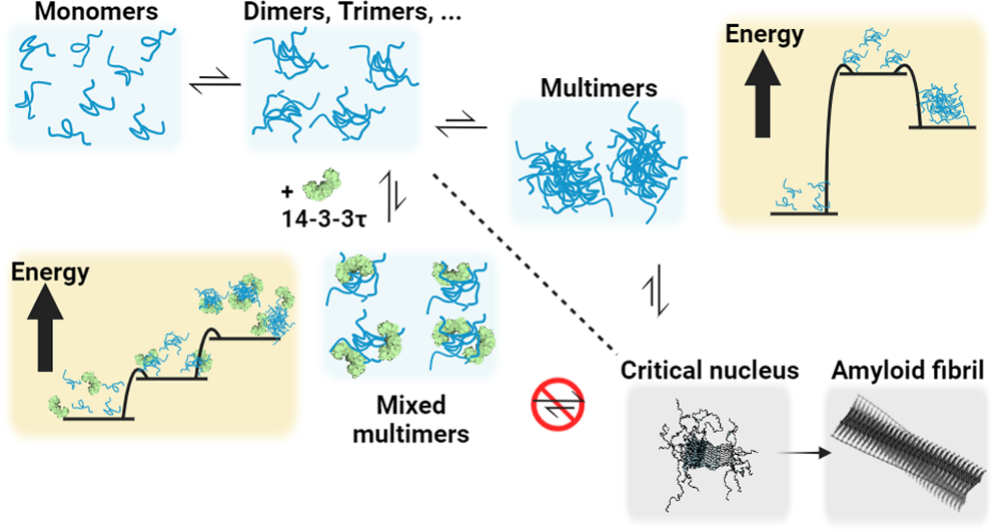

The aggregation of α-synuclein (αS) plays a key role in Parkinson’s disease (PD) etiology. While the onset of PD is age-related, the cellular quality control system appears to regulate αS aggregation throughout most human life. Intriguingly, the protein 14-3-3τ has been demonstrated to delay αS aggregation and the onset of PD in various models. However, the molecular mechanisms behind this delay remain elusive. Our study confirms the delay in αS aggregation by 14-3-3τ, unveiling a concentration-dependent relation. Utilizing microscale thermophoresis (MST) and single-molecule burst analysis, we quantified the early αS multimers and concluded that these multimers exhibit properties that classify them as nanoscale condensates that form in a cooperative process, preceding the critical nucleus for fibril formation. Significantly, the αS multimer formation mechanism changes dramatically in the presence of scaffold protein 14-3-3τ. Our data modeling suggests that 14-3-3τ modulates the multimerization process, leading to the creation of mixed multimers or co-condensates, comprising both αS and 14-3-3τ. These mixed multimers form in a noncooperative process. They are smaller, more numerous, and distinctively not on the pathway to amyloid formation. Importantly, 14-3-3τ thus acts in the very early stage of αS multimerization, ensuring that αS does not aggregate but remains soluble and functional. This offers long-sought novel entries for the pharmacological modulation of PD.

## Introduction

α-Synucleinopathies, such as Parkinson’s disease (PD), dementia with Lewy bodies, and multiple system atrophy, are characterized by the accumulation of fibrillar α-synuclein (αS) aggregates in brain cells.^1^ αS, a 140 amino acid protein, is involved in membrane remodeling and trafficking in the brain.^2−4^ Its dysfunction leads to aggregation into amyloid fibrils, which deposit in specific brain cells and areas.^5^ The aggregation of αS starts a toxic cascade; once the first fibrils are formed, they become the catalyst for further aggregation, perpetuating a cycle that spreads pathology from cell to cell.^1,6^ Understanding how this aggregation process is held in check in healthy cells presents a significant challenge.^6^

The chaperone proteins of the cellular protein quality control system play an important role in maintaining proteostasis. Intriguingly, already formed αS amyloid fibrils can be disaggregated by the adenosine 5′-triphosphate (ATP)-dependent HSP70 chaperone system.^7^ However, this intervention is akin to damming a river after the flood; the disaggregation process may inadvertently release seeding-prone fragments, which can contribute to further propagation of the disease state.^8^ Disaggregating fibrils is therefore, most likely a backup plan, not the primary line of cellular defense against toxic aggregation. This highlights the importance of earlier intervention in the aggregation process, particularly at the oligomer and condensate stages, where chaperones like proteins from the 14-3-3 protein family are suspected to play a vital role.^9,10^

The 14-3-3 protein family is a group of highly conserved adapter proteins consisting of seven human isoforms (β, γ, ε, η, τ/θ, ζ, and σ).^11^ With hundreds of protein interaction partners, the 14-3-3 family plays a major regulatory role in the human cell.^12−15^ The 14-3-3 proteins mainly recognize phosphoserine and phosphothreonine motifs, but phosphorylation-independent interactions have also been reported.^16^ Generally, the 14-3-3 binding proteins interact via the amphipathic binding groove of 14-3-3, additionally the last two C-terminal α-helices of 14-3-3 have been demonstrated to contribute to interactions.^11^

14-3-3s constitute 1% of all soluble proteins in the brain, and decreasing levels of 14-3-3 expression, either due to downregulation or due to age-related decline, have been related to neurodegenerative protein aggregation diseases.^17−20^ Several studies suggest that interactions between αS and 14-3-3 proteins mitigate αS oligomerization and toxicity but also obstruct the seeding and internalization of αS aggregates in systems ranging from in vitro constituted proteins to animal models.^9,21,22,10,23^ Notably, 14-3-3 has been found to even effectively reduce αS toxicity, when present in low substoichiometric ratios.^9,23^ The findings indicate that 14-3-3 proteins might serve as protective agents against protein aggregation, potentially providing a defense against the onset of α-synucleinopathies. However, the exact ways in which 14-3-3 proteins counteract protein aggregation are still not well understood.

Investigating the interaction between early αS multimers and 14-3-3 is challenging due to the transient and rare nature of αS multimers. To address the challenges in studying early αS aggregation, we utilized microscale thermophoresis (MST) and single-molecule (SM) fluorescence burst analysis. Combined with MST, these techniques allowed us to directly observe the process of αS multimerization. Our investigations compellingly demonstrate that αS proteins cooperativly assembly into dynamic multimers at a critical concentration, marking a significant stride in our understanding of αS aggregation. The αS multimers exhibit characteristics of nanoscale condensates and are formed on pathway to the critical nucleus for αS fibril formation. In light of the observed delay of αS aggregation and toxicity in the presence of the 14-3-3τ isoform, we strategically chose this isoform for our in-depth analysis. We show that the delay in αS aggregation is directly tied to the interactions between 14-3-3τ and dynamic αS multimers. Our findings reveal that 14-3-3τ actively modulates the αS multimerization. In the presence of 14-3-3τ, the multimerization process of αS is dramatically altered, transitioning from a cooperative to a noncooperative assembly mode and leading to the formation of mixed αS/14-3-3τ multimers. These emergent co-condensates play a crucial role, diverting αS away from forming critical nuclei essential for fibril formation. Consequently, this interaction significantly postpones the aggregation of αS into amyloid fibrils. This discovery not only advances our understanding of αS aggregation but also may open new avenues for pharmacological interventions in PD and related neurodegenerative diseases.

## Materials and Methods

### Protein Production

Recombinant human αS wild-type (αS-WT), αS-A140C, and αS–S42C-A90C mutants were expressed in *Escherichia coli* BL21 (DE3) cells using the pT7–7 expression system. A detailed protocol for expression and purification is available in the mentioned reference.^24^ The 14-3-3τ protein was expressed as described in the specified literature.^25^ Following purification, proteins were aliquoted, flash frozen, and stored at −80 °C in 10 mM Tris (pH 7.4). Prior to the experiments, samples were thawed freshly. Solutions containing the cysteine mutants were supplemented wih 1 mM dithiothreitol (DTT).

### Protein Labeling

αS-A140C was labeled with Alexa Fluor 488 (AF488, Thermo Fisher Scientific) via a maleimide–thiol reaction, in accordance with the manufacturer’s guidelines. 14-3-3τ was labeled by targeting its primary amines using NHS-ester functionalized Alexa Fluor 568 (AF568, Thermo Fisher Scientific) according to the manufacturer’s protocol. Subsequently, αS-A140C-AF488 (αS488) and 14-3-3τ-AF568 conjugates were purified by using a 7 kDa MWCO Zeba Spin desalting column (Pierce Biotechnology). The label-to-protein ratio, the degree of labeling (DOL), was assessed using UV–vis absorption (NanoDrop, Thermo Fisher Scientific), resulting in a 1:1 DOL for αS488 and a 1:7 DOL for 14-3-3τ-AF568 (i.e., not all proteins are labeled).

To label αS–S42C-A90C with both AF488 and AF568 (αS488/568), potential disulfide bonds in the protein were initially reduced with 1 mM DTT. Post desalting with a 7 kDa MWCO Zeba Spin (Pierce Biotechnology), an equimolar concentration of AF488 was introduced and incubated for 30 min at room temperature (RT), followed by desalting using a Hitrap desalting column (Cytiva Life Sciences). BcMag thiol-activated magnetic beads (Bioclone Inc.) were used to bind single-labeled and unlabeled αS–S42C-A90C, thereby removing the double-labeled αS. The bead-bound protein was eluted using 100 mM DTT in 100 mM NaPO_4_ (pH 7.4), concentrated, and subsequently mixed with an excess (1:1.4 molar ratio) of AF568. After incubating for 2 h at RT, UV–vis absorption was used to quantify protein and label concentrations.

Labeled proteins were aliquoted, flash frozen, stored at −80 °C, and freshly thawed before the experiments.

### 30-mer Formation

αS_30_ oligomers were generated as detailed in the specified protocol.^26^ Briefly, αS was incubated at a high monomer concentration (>1 mM) for 18 h at RT with gentle shaking (300 rpm). This was followed by a 2 h incubation at 37 °C without shaking. The αS_30_ was then purified using a size-exclusion column (Superdex 200 Increase 10/300 GL, Sigma-Aldrich, UK). A method similar to that was used for αS488/568-doped αS_30_ (αS_30_-488/568) production. However, αS488/568 was added to αS-WT at a final 1/30 molar fraction, resulting in approximately 1 αS488/568 per αS_30_ on average.

### Aggregation Assay

αS aggregation assays were conducted on an Infinite 200Pro plate reader (Tecan Ltd., Switzerland). Assays were carried out in 96-well half-area clear flat-bottom, untreated polystyrene microplates (3695, Corning). The assay conditions were incubation of αS at 37 °C with shaking at 432 rpm, in the presence or absence of 14-3-3τ, in 10 mM Tris (Sigma-Aldrich, U.K.) (pH 7.4), 10 mM NaCl (Sigma-Aldrich), 10 mM ThT (Fluka, Sigma-Aldrich, U.K.), and 0.02 w/v% NaN_3_. The specific protein concentrations are specified in the main text. Samples were prepared in triplicate with a volume of 150 μL. Every 10 min, without shaking, the samples were excited at 446 nm to monitor β-sheet content by ThT emission intensity at 485 nm. The definition of the lag time is detailed in the provided section.

### Thermal Shift Assay (TSA)

Thermal shift assays were performed using 40 μL samples containing 2.5 μM 14-3-3τ and 25 μM αS or ERα with 10x ProteoOrange (Lumiprobe, 5000x stock in DMSO) in 10 mM Hepes, 150 mM NaCl, and 50 μM TCEP (pH 7.4). The samples were heated from 35 to 79 °C at a rate of 0.3 °C per 15 s in a CFX96 Touch Real-Time PCR Detection System (Bio-Rad). Fluorescence intensity was determined using excitation and emission filters of 525/20 and 570/20 nm, respectively. Based on these melting curves, the negative derivative melting curve is obtained, from which the melting temperature *T*_m_ was determined. All described melting temperatures are based on three independent experiments performed in duplicate. The change in the melting temperature, Δ*T*_m_, is determined per experiment.

### Self-Assembly Model

The used self-assembly model^27^ describes the total protein concentration *c*_*t*_ as a function of the free monomer concentration [*A*], the equilibrium constant of any monomer addition step *K*, with *K*^–1^ = *c*_c_, where *c*_c_ is the critical concentration, and a cooperativity factor σ defined as σ = *K*_*N*_/*K*, where *K*_*N*_ is the equilibrium constant for nucleus formation (in case of a single step, *N* = 2).
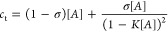
1Using eq 1, we describe the free monomer fraction [*A*]/*c*_t_ as a function of the total protein concentration *Kc*_t_. We use *Kc*_t_ instead of *c*_t_ for the generalization of the model.^27^ To compare the model with measured data, we plot [*A*]/*c*_t_ as a function of *c*_t_. Additionally, because the MST response (*F*_norm_) is not directly a quantitative read-out, we consider the monomer fraction to be relative, i.e., although ranging from 0 to 1, [*A*]/*c*_t_ only represents the changes in the population of two equilibrium states. Note that the cooperativity factor σ decreases when the cooperativity of the assembly process increases.

### Microscale Thermophoresis Measurements and Analysis

MST measurements were conducted using a Monolith NT.115 instrument (NanoTemper). Experiments were performed in a solution containing 10 mM Tris buffer (pH 7.4) with 10 mM NaCl at 37 °C unless stated otherwise. A labeled protein concentration of 50 nM was used unless indicated differently. The labeled proteins, αS488 and 14-3-3τ-AF568, were excited using the blue and green light-emitting diode (LEDs) of Monolith NT.115, respectively. The excitation power was adjusted to achieve an approximate fluorescence emission intensity of 1000 au. Potential biases, such as protein adsorption to capillaries, were assessed and ruled out by examining capillary scans.^28^

The MST response, denoted as *F*_norm_, was computed by comparing the fluorescence intensity prior to and following infrared (IR) laser activation, termed *F*_cold_ and *F*_hot_, respectively. The time point at which the IR laser was turned is defined as *t* = 0 s. We determine the intensity level of *F*_cold_ from the average intensity between *t* = −1 to 0 s and *F*_hot_ from *t* = 5 to 30 s. The MST responses were measured at two distinct IR-laser intensities, 60 and 80%, and their results were averaged. Variability in the MST response, represented as error bars, was derived from early (*t* = 3–5 s), mid (*t* = 12–14 s), and late (*t* = 28–30 s) intervals for both IR laser intensities and presented as the standard deviation (STD).

Measurements of αS multimerization, both in the presence and absence of 14-3-3τ, were executed using the same dilution series. To study heteromolecular interactions in MST experiments, *F*_hot_ was determined from the average intensity during *t* = 29–30 s. Measurements with 14-3-3τ-AF568 were performed at 23 °C. When examining αS_30_, where the maximum concentration that could be reached was 2.1 μM (equivalent to 63 μM of monomer), the concentration contrast between 14-3-3τ and αS_30_ was increased by reducing the 14-3-3τ-AF568 concentration to 7 nM, yielding an overall 50 nM 14-3-3τ concentration (DOL = 1/7). These samples were subjected to a 15 h incubation at room temperature (RT) prior to measurements at 25 °C. The IR-laser intensities used for these measurements were 40, 60, and 80%.

Raw data were processed using the MO.Affinity Analysis software by NanoTemper. Interaction curves for heteromolecular interactions were fitted using the provided *K*_D_ and Hill models, as outlined by Wienken et al.^29^ and Scheuermann et al.^30^ The αS multimerization data was analyzed in Matlab2020b using a (nucleated) self-assembly model (eq 1^27^). For the fitting, MST responses were first normalized based on the initial and final plateaus, resulting in values spanning between 1 (indicative of a “monomer-only” state) and 0 (representing a combination of “monomer and multimer” states). Given that the critical concentration (*c*_c_) and cooperativity factor (σ) are interrelated parameters in distinct dimensions, *Kc*_t_ and [*A*]/*c*_t_, respectively, we used combinations of *c*_c_ and σ. For each set, the error between the experimental data and the model was assessed. The combination yielding the smallest error was deemed the best fit. To avoid a bias introduced by an outlier in the data, this fitting process was executed in two stages. In the initial stage, the data point at low *c*_t_ exhibiting the greatest deviation from the fit curve was identified as an outlier and was subsequently excluded during the second fitting iteration. We confirmed the robustness and reproducibility of the fits (SI, Figures S5 and S6).

### Single-Molecule Burst Detection and Analysis

SM fluorescence techniques, including single-molecule fluorescence burst analysis, have been used successfully to obtain insight into molecular mechanisms.^31,32^ Single-molecule fluorescence burst experiments were carried out by using a commercially available confocal microscope (PQ-MT200). The labeled αS (αS488) was excited by using a 485 nm laser (PicoQuant, LDH-485-D-C) operating at a pulse rate of 10 MHz and delivering an optical power of 20–25 μW in the back focal plane. A dichroic mirror (Chroma, ZT488/561rpc-uf3) was used to direct the excitation light toward the microscope’s objective (Olympus, UPLSAPO60XW 1.2 NA), focusing the light to a diffraction-limited volume, the detection volume. The subsequent emission was collected via the same objective, spatially filtered by a 100 μm pinhole, spectrally filtered using a band-pass filter (Semrock, FF01-520/35), and detected by a single-photon avalanche detector (SPAD, Excelitas SPCM-AQRH-14-TR). Each detected photon was time-stamped and related to the corresponding excitation pulse with time-correlated single-photon counting (TCSPC), generating time-tagged time-resolved (TTTR) fluorescence data.

For the experiments, αS488 was diluted to picomolar concentrations, aiming for 1–10 isolated fluorescence bursts per second. The final sample mixtures were composed of (1) αS488 only, (2) αS488 with 110 μM αS-WT, and (3) αS488 with 110 μM αS-WT and 10 μM 14-3-3τ. Every sample was prepared in 10 mM Tris buffer (pH 7.4) containing 10 mM NaCl. Five μM αS-WT was applied to passivate the cover glasses. The cover glasses were subsequently rinsed with buffer solution, and the samples were placed on the αS-coated cover glasses. Measurements were executed in solution, approximately 30 μm above the glass-solution interface. For each sample, three-time traces of 1800 s each were recorded, accumulating approximately 12k bursts per measurement (equivalent to ≈6 bursts/s).

Bursts were identified using custom-written Python code. The identification was based on a sliding window burst search algorithm as described in ref (33). The parameters for burst detection included a brightness threshold, a minimum photon count of 10 per burst, and a minimum separation of 160 μs between consecutive bursts. Bursts with separations <160 μs were merged. The passage times are determined by the difference in absolute arrival time between the initial and final photon of the identified burst.

## Results

### Delay of αS Aggregation by 14-3-3τ

**Figure 1:**
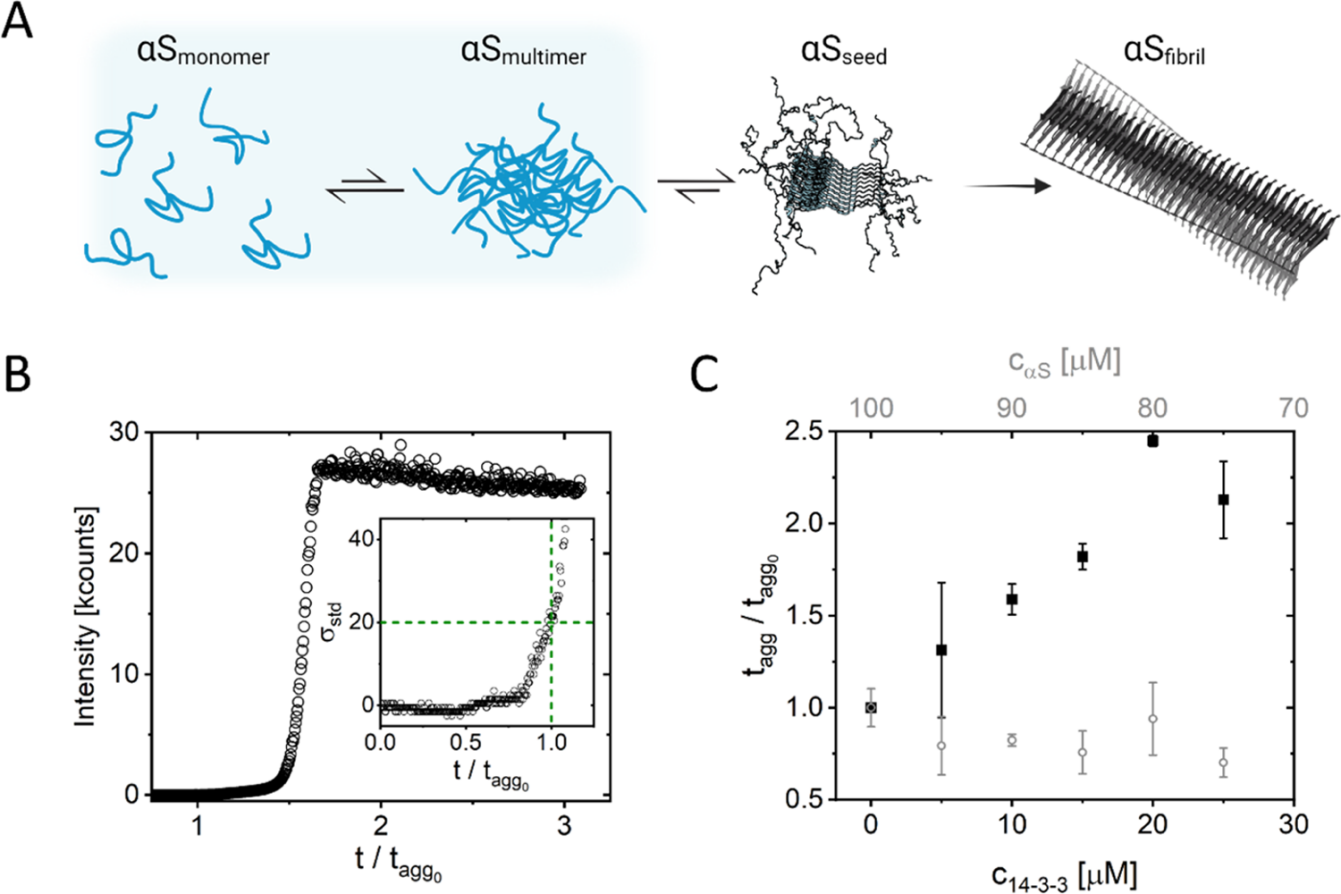
(A) Simplified schematic depicting the process of αS aggregation into amyloid fibrils. Monomers assemble into multimers, and some of these multimers develop into the critical nucleus for amyloid formation from which finally amyloid fibrils appear. (B) Typical αS aggregation curve obtained in the absence of 14-3-3τ. Aggregation was monitored by following the increase in the ThT intensity. In this graph, the time is given relative to the lag time in the absence of 14-3-3τ (*t*_agg_0__). The lag time is defined as the time at which the background corrected intensity exceeds 20 times the standard deviation (STD) of the initial ThT intensity. The inset shows a zoom of (B), but now, the intensity is given relative to the STD (σ_std_). (C) Increase of the lag time with increasing concentration of 14-3-3τ (black, closed symbols) at a constant αS concentration of 100 μM. To exclude that the change in lag time is due to the sequestering of monomers, we present an αS concentration series as a control (gray, open symbols).

The protein αS aggregates into amyloid fibrils via multimeric intermediates (Figure 1A). The 14-3-3 proteins and, in particular, the 14-3-3τ isoform, have previously been shown to reduce αS aggregation and toxicity.^9,23,34^ Hence, we selected this isoform for our studies. To quantify the effect of 14-3-3τ on αS aggregation, we performed standard ThT assays to monitor the increase in αS fibril mass with time at increasing 14-3-3τ concentrations. In the standard ThT assay, the time to the onset of aggregation is referred to as the lag time (Figure 1B). With increasing 14-3-3τ concentration, the lag time increases (Figure 1C, SI Figure S1). To test if this increase in lag time results from interactions of 14-3-3τ with αS monomers, which would decrease the concentration of αS monomers available for aggregation, we also performed experiments at reduced αS concentrations. In the αS concentration range tested, the aggregation lag time did not increase. We, therefore, conclude that the increase in aggregation lag time is not a result of a reduction in the αS monomer concentration available for aggregation. In agreement with this finding, we do not find any signs of interactions between 14-3-3τ and αS monomers in a thermal shift assay (TSA) (SI, Figure S2). In the absence of interactions between αS monomers and 14-3-3τ, the delay in aggregation must be due to an interaction of 14-3-3τ with multimeric αS species that appear before the critical nucleus required for fibril formation is formed (Figure 1A, αS_multimer_). We, therefore, conclude that 14-3-3τ influences the multimerization on the pathway toward a critical nucleus for fibril formation.

### Monitoring αS Multimers

Studying αS self-assembly into multimers is not trivial. The multimerization process is energetically unfavorable, and therefore, only a low fraction of the monomers will assemble into multimers; the multimers of interest will be rare. We were, for example, not able to capture multimeric species in gel electrophoresis experiments. This suggests that multimers are not only rare but also dynamic in nature; there is an exchange of αS between multimers and solution. Both their rarity and dynamic nature complicate the study of multimers.

**Figure 2:**
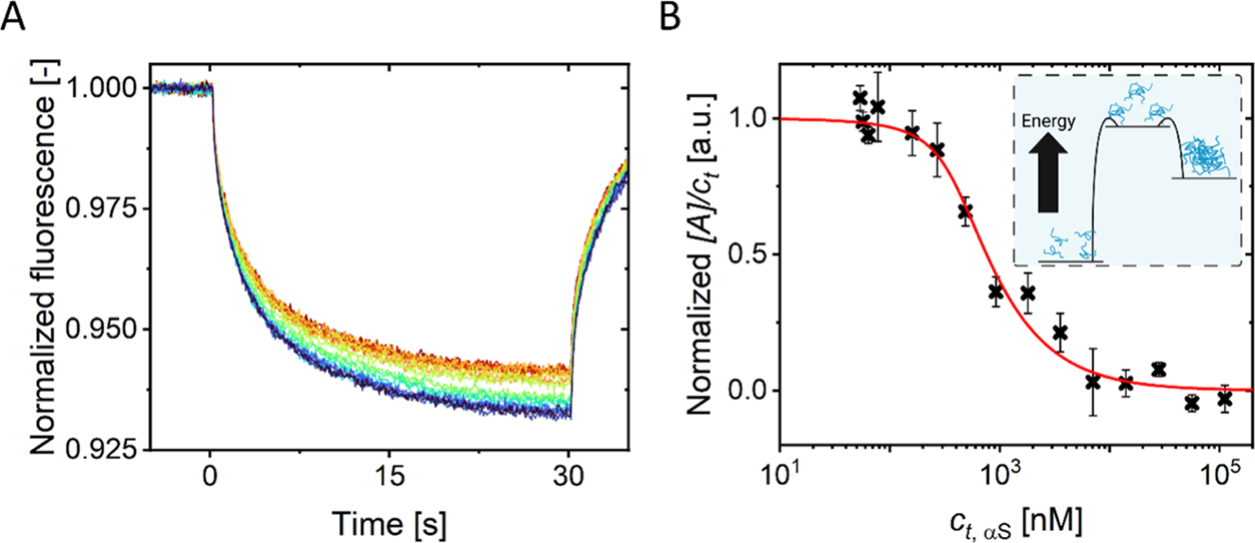
(A) Normalized fluorescence response in an MST experiment probing αS multimerization. Unlabeled αS was added at increasing concentrations from 3 nM (blue) to 112 μM (red) to a constant concentration of fluorescently labeled αS (50 nM) (B) Self-assembly of αS multimers. Normalized MST response of labeled αS as a function of total αS concentration. The systematic change from the plateaus at low and high total αS concentrations indicates early multimerization of αS. This curve is fitted by a self-assembly model (eq 1). This fit results in parameters σ = 0.06 & *c*_c_ = 0.5 μM. The low value of the cooperativity factor σ clearly demonstrates that early-stage αS multimerization is a nucleation-dependent process. Note that we plot [*A*]/*c*_t_ as a function of *Kc*_t_*c*_c_ = *c*_t_, where *c*_c_ follows from the fit. The inset shows a schematic depicting the energy landscape associated with early αS multimerization derived from the presented data.

To follow the self-assembly process of αS into dynamic multimers, we performed MST experiments (Figure 2A). In these experiments, we used a constant, low concentration of fluorescently labeled αS while we increased the concentration of unlabeled αS. With increasing αS concentration, the MST response systematically changed from a plateau at low αS concentrations to a second plateau at increasing total αS concentration (Figure 2B). The change in the MST response suggests that αS self-assembles into multimers at higher αS concentrations. The inflection point in the self-assembly curve was found at approximately 0.5 μM.

**Figure 3:**
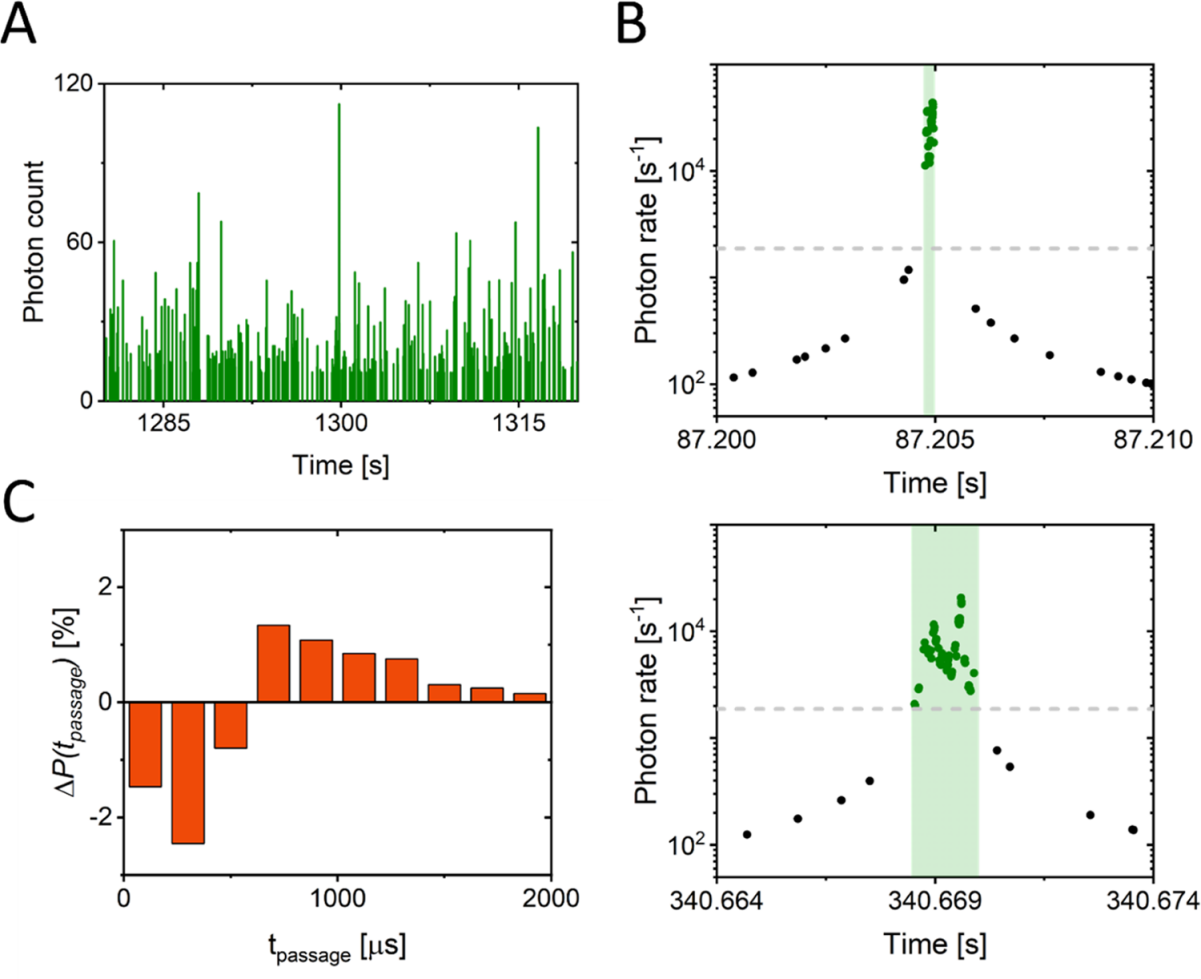
(A) Typical fluorescence burst trace of labeled αS at a picomolar concentration. (B) Example of fluorescence burst duration (passage time) detection, depicting a short (top) and a long (bottom) burst. Note that the length of the *x*-axis is equal in both panels. The gray-dashed line indicates the burst brightness detection threshold. (C) Difference in normalized passage time distributions between high and low total αS concentration. The difference is given in percent change. At short *t*_passage_, we observe a negative difference when comparing low to high αS concentrations; at longer *t*_passage_, we observe a positive difference. Quantification of the observed shift indicates that approximately 5% of the αS monomers are present as multimers at high αS concentrations.

To verify the presence of self-assembled multimeric αS species at higher αS concentrations and to determine the fraction of αS monomers that are assembled into these species, we performed single-molecule burst (SM burst) experiments (Figure 3A). In SM burst detection, we use the direct relationship between size and diffusion speed to distinguish between monomers and multimers. For individual, fluorescently labeled monomers and multimers, we determined the passage time through the optical detection volume. The passage time is not constant but distributed due to the random nature of diffusion through the detection volume. We determined the individual passage times from the measured burst durations (Figure 3B). The passage times of the individual monomers and multimers were accumulated in a normalized histogram. Passage time histograms were obtained for two conditions corresponding to the plateaus in Figure 2B: (1) a monomer-only condition characterized by low total αS concentration and (2) a monomer + multimer condition associated with high total αS concentration (SI, Figure S3). We used picomolar concentrations of labeled αS in both conditions to ensure the presence of less than one fluorescently labeled monomer or multimer in the detection volume. The use of a low ratio between labeled and unlabeled αS (∼1:10^7^) ensured that formed multimers contained at most one labeled αS monomer and thus the measured changes in passage time directly reported on the fraction of multimers. For both conditions, we calculate the average passage time (SI, Figure S4). We found an increase from 639 ± 9 μs at low αS concentration where only αS monomers are present to 681 ± 6 μs at high αS concentration where we expect multimers to form. Overall, the change in diffusion time seems small. However, note that only a small fraction of the αS monomers are expected to assemble into multimers; hence, the contribution of the multimers to the average is small.

To quantify the fraction of the labeled αS in multimers, we subtracted the normalized histograms for condition 1 from condition 2, defined as Δ*P*(*t*_passage_) (Figure 3C). At high αS concentrations, we observed a decrease in the fraction of αS present as monomers (short passage times up to 600 μs) and an increase in the fraction of monomers in multimers (long passage times >600 μs). Quantitatively, we found that approximately 5% of the labeled αS proteins are present in the slower diffusing multimers; hence, 5% of the observed bursts originate from multimers. Note that the resolving power of differently sized species is limited due to the relatively wide distribution of passage times, and hence, this fraction is a lower limit. There may be multimers of low aggregation numbers that cannot be discriminated from monomers in these experiments.

Summarizing, at concentrations above approximately 0.5 μM, αS self-assembles into multimers. Since the time scale on which amyloid fibrils appear (typically days under the conditions used) is orders of magnitude larger, the formed αS multimers are not yet the critical nucleus for fibril formation. The multimers are probably metastable intermediates on the pathway to amyloid formation.

### Interaction between 14-3-3τ and αS

**Figure 4:**
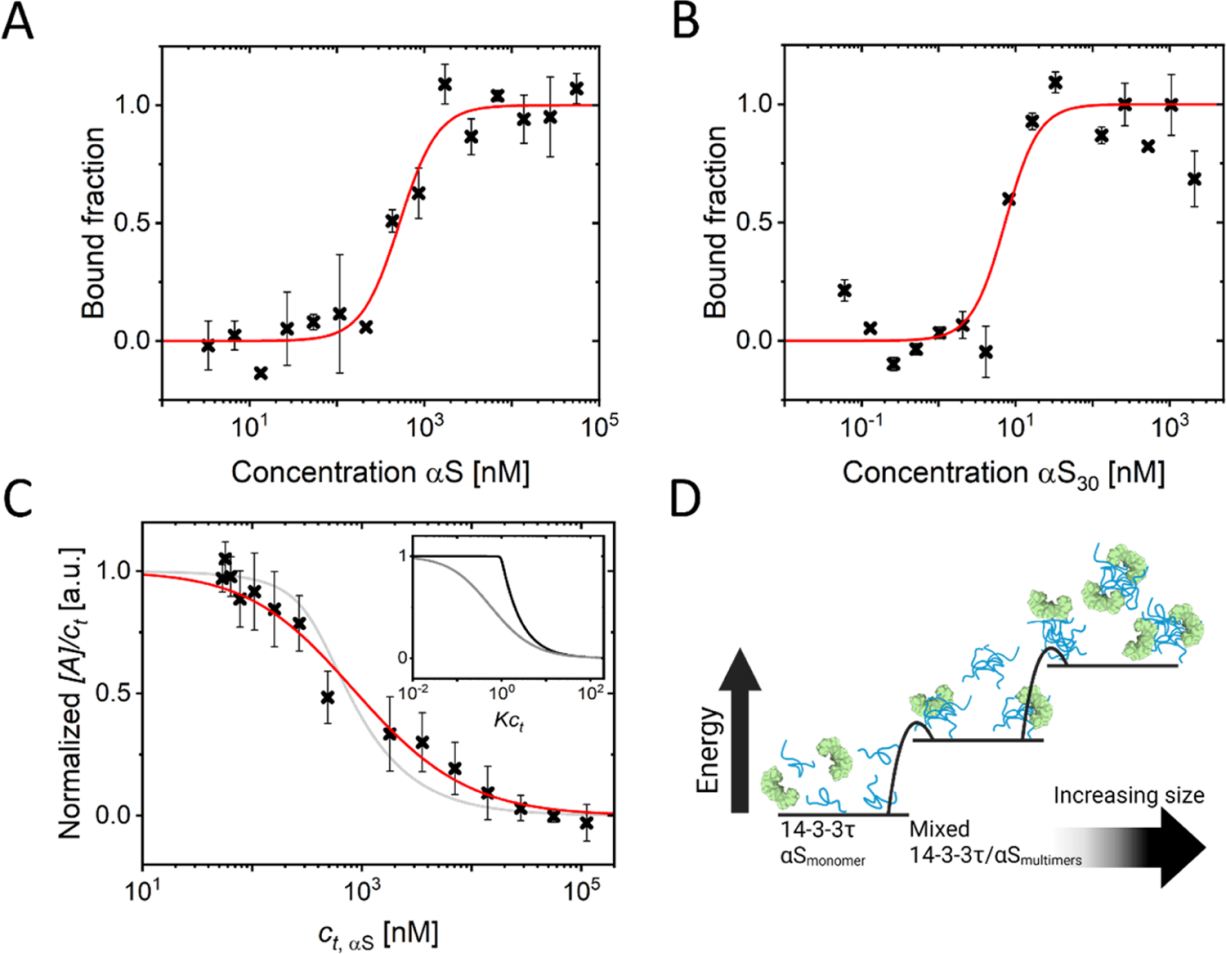
(A) Heteromolecular interaction between 14-3-3τ and αS. The interaction was probed using MST on a solution of 338 nM total 14-3-3τ of which 50 nM was labeled (14-3-3τ-AF568) at increasing concentrations of αS. The inflection point of the interaction curve lies at ∼0.5 μM αS. The data was fitted to the Hill equation (red line) with *n*_Hill_ = 2, yielding EC_50_ = 519 nM under the conditions used, assuming that the 14-3-3τ interacts with multimers as explained in the main text. (B) Heteromolecular interaction between 14-3-3τ and αS_30_. The interaction was probed using MST on a solution of 7 nM 14-3-3τ-AF568 (50 nM total 14-3-3τ) at increasing concentrations of αS_30_. Note: the *x*-axis shows the αS_30_ concentration, not the monomer equivalent concentration. The data was fitted to the Hill equation (red line) with *n*_Hill_ = 2, yielding EC_50_ = 7 nM for the conditions used. The error bars in parts (A) and (B) represent the standard deviation from measurements performed at different IR-laser powers. (C) Self-assembly of αS multimers in the presence of 14-3-3τ. Normalized MST response at a 50 nM αS488 as a function of total αS concentration in the presence of 3.5 μM 14-3-3τ. The curve is fitted to a nucleated self-assembly model (eq 1), resulting in parameters σ = 1.26 and *c*_c_ ≈ 1778 nM. For comparison, the fit from Figure 2B (αS multimerization in the absence of 14-3-3τ) is plotted in light gray. The inset, with the same *y*-axis, shows strong nucleation (σ = 1e-4 and *c*_c_ = 224 nM) and no nucleation (σ = 1 and *c*_c_ = 1585 nM) model curves in black and gray, respectively, clearly demonstrating the loss of nucleation in αS multimerization when 14-3-3τ is present (D) Schematic depicting the energy landscape associated with αS multimerization in the presence of 14-3-3τ as derived from the presented data.

To gain further insights into how the interaction between 14-3-3τ and αS multimers delays αS aggregation (Figure 1A), we started with MST experiments. We measured the MST response of a constant concentration labeled 14-3-3τ (AF568) in the presence of increasing αS concentrations, spanning the concentration regime where we observe αS multimerization (Figure 2B). Figure 4A shows a constant MST response at αS concentrations where multimers were absent, while the MST response changes and plateaus at the higher concentrations where we observed αS multimerization. The changes in the MST response occurred in the αS concentration regime where we observe αS multimerization, which suggests that 14-3-3τ and αS multimers interact. The inflection point of the MST response curve lies at an αS concentration of ∼0.5 μM. This is in good agreement with the inflection point observed for αS multimerization, which suggests a high affinity between 14-3-3τ and αS multimers. Since this interaction of 14-3-3τ with αS multimers increases the aggregation lag time, we conclude that the αS multimers observed in MST experiments in the absence of 14-3-3τ are a metastable intermediate, on the pathway to the formation of amyloid fibrils.

To confirm the high affinity between 14-3-3τ and αS multimers, we estimate the concentration of the multimers. Our estimation is based on the fraction of monomers in multimers (Figure 3C) and an estimation for the number of monomers in a multimer. To estimate the number of monomers in a multimer, we use the passage time of the multimers observed in SM burst analysis. For the multimers, we observe passage times longer than 600 μs (Figure 3C). For comparison, we measured the passage time of a relatively stable αS species that appears at elevated αS concentrations.^26,35−37^ Contrary to the metastable multimers, this species is sufficiently stable to isolate. It has been characterized in different laboratories using small-angle X-ray scattering (SAXS), HDX-MS, nuclear magnetic resonance (NMR), and photobleaching experiments, resulting in a consensus that this αS species is largely unstructured and contains ∼30 monomers.^36,38−40^ We refer to this species as αS_30_. For this αS_30_ species, we determined the passage time to be approximately 900 μs (SI, Figure S7). Comparing passage times, we conclude that the metastable multimers we observe in our experiments consist of a relatively low number of monomers comparable to the αS_30_ species but likely slightly less. For a back-on-the-envelope calculation to estimate the affinity, we assume that they consist of 5–20 monomers, which translates into a binding affinity between 14-3-3τ and αS multimers in the order of a few nanomolar.

Since the αS_30_ has been found to be largely unstructured and the number of monomers in this species is comparable to that of the metastable αS multimers, we expect that 14-3-3τ will also interact with this species. To confirm this, we measured the MST response of a fixed concentration of labeled 14-3-3τ as a function of increasing αS_30_ concentrations. Plotting the normalized MST response (or equivalent, the fraction bound) as a function of the αS_30_ concentration resulted in the expected binding curve (Figure 4B). To obtain an estimate for the affinity between 14-3-3τ and αS_30_, we fitted the MST response to the Hill equation, which gives an EC_50_ value of approximately 7 nM with a Hill coefficient of 2. We, therefore, conclude that there is a strong, cooperative interaction between 14-3-3τ and αS_30_. This affinity agrees well with the estimated interaction strength for the metastable multimers. We, hence, conclude that 14-3-3τ indeed interacts with αS multimers with high affinity. Moreover, since 14-3-3τ interacts with both the metastable αS multimers and the stable αS_30_ species, the interaction is not limited to a specific αS multimer species or size; the interaction is more generic.

### Impact of 14-3-3τ on αS Multimerization

After having established that 14-3-3τ and αS multimers interact with high affinity, we investigated whether 14-3-3τ modifies the multimerization process and thereby delays the formation of a critical nucleus that can grow into amyloid fibrils.

We performed MST experiments to monitor αS multimerization as previously described in the presence of 3.5 μM 14-3-3τ. The results are plotted in Figure 4C. We observe a clear change in the MST response; while the normalized MST response started to decrease at lower concentrations and the overall response was less steep, the inflection point was found at a comparable αS concentration. Note that the concentration is plotted on a logarithmic scale, and the multimerization features were drastically altered by the presence of 14-3-3τ.

### Modeling the Multimerization of αS in the Absence and Presence of 14-3-3τ

To understand the altered multimerization of αS at a mechanistic level, we fitted the MST multimerization curves, assuming multimerization occurs by nucleated self-assembly^27^ (see Materials and Methods). The model for nucleated self-assembly is easily adapted to various situations, including an isodesmic and a cooperative assembly process.^27^ The model eq 1 includes both a cooperativity factor σ, representing the prominence of the cooperativity, and a critical concentration *c*_c_, signifying the concentration at which multimers start forming. Fitting the MST αS multimerization data in the absence of 14-3-3τ (Figure 2B), we find good agreement between the MST data and the model for σ ≈ 0.06 and *c*_c_ ≈ 0.5 μM (red line in Figure 2B). We, therefore, conclude that the self-assembly of the dynamic metastable αS multimers is a cooperative process that is characterized by a critical concentration (Figure 2B, inset). Fitting the MST αS multimerization data in the presence of 3.5 μM 14-3-3τ gives σ ≈ 1.26 and *c*_c_ ≈ 1.8 μM (Figure 4C). We, therefore, conclude that 14-3-3τ is changing the multimerization process itself (Figure 4D), and this effect was not observed in a negative control sample (SI, Figure S8). Note that the onset of the transition from monomers to multimers with concentration is abrupt in a highly cooperative model (σ approaches zero) and more gradual in a noncooperative situation (σ ≥ 1) (Figure 4C, inset).

**Figure 5:**
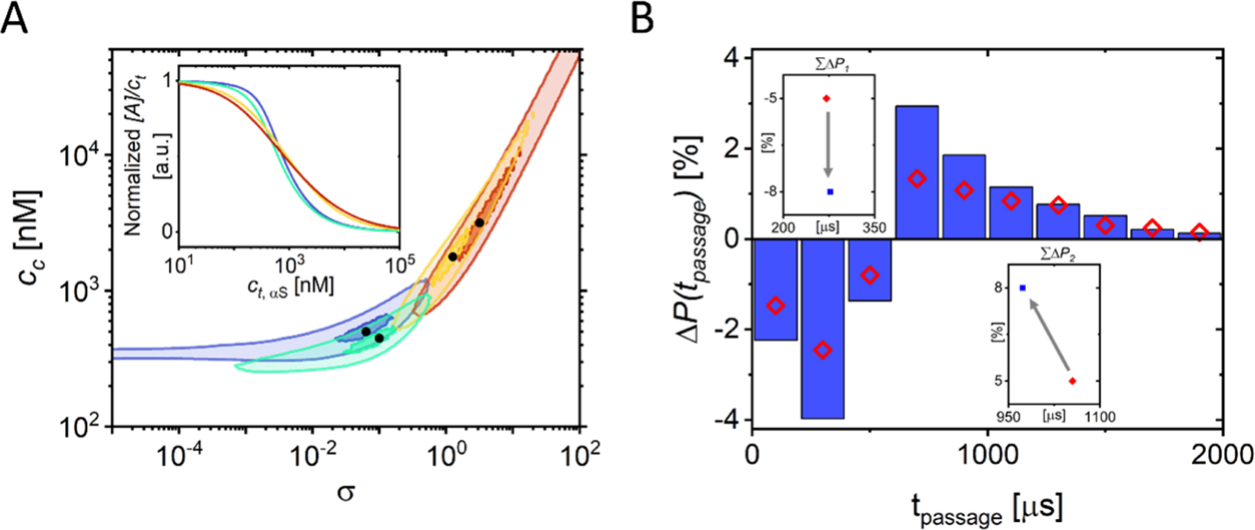
(A) Best model fits of σ and *c*_c_ to the MST data at increasing 14-3-3τ concentrations. Confidence intervals corresponding to 0.5th (small shaded area) and 3rd (large shaded area) percentiles of the best fits are given. The inset shows the best-fit curves of αS multimerization in the absence (blue) and presence of 875 nM, 3.5 μM, and 7 μM 14-3-3τ (green, yellow, and red, respectively). (B) Difference in normalized passage time distributions between high and low total αS concentrations (Δ*P*(*t*_passage_)) in the presence (blue bars) and absence of 14-3-3τ (red diamonds). In determining the difference, the total number of bursts is accounted for, and the difference is given in percent change. At short *t*_passage_, we observe a negative difference, and at longer *t*_passage_, we observe a positive difference. Comparing multimerization in the absence (red diamonds) and presence of 14-3-3τ (blue bars), we observe that in the presence of 14-3-3τ, more monomers are present in multimers. Quantifying the shift from short passage times (<600 μs) to longer passage times (>600 μs) gives 5% multimers in the absence of 14-3-3τ and 8% multimers in the presence of 14-3-3τ. Also, in the absence and presence of 14-3-3τ, the average passage time of short bursts is ≈271 and ≈277 μs (see inset ∑Δ*P*_1_) and that of long bursts is ≈1055 and ≈973 μs (see inset ∑Δ*P*_2_), respectively. We, therefore, conclude that multimers formed in the presence of 14-3-3τ are on average smaller but more numerous.

To monitor how different concentrations of 14-3-3τ affect the αS multimerization process, we obtained additional MST data on αS multimerization in the presence of 875 nM and 7 μM 14-3-3τ. This data was also fitted to eq 1. All of the obtained values for σ and *c*_c_ are plotted in Figure 5A, where we show the best fit and the 0.5th and 3rd percentiles of the best fits to the MST data. Both the best fits and the 0.5th and 3rd percentile of the best fits show a clear trend toward an increase in the cooperativity factor σ and hence a decrease and eventually a loss of cooperativity with increasing 14-3-3τ concentration. At the same time, *c*_c_ also increased with the 14-3-3τ concentration. Note that with increasing 14-3-3τ concentration, not only the value but also the uncertainty in *c*_c_ increases as σ increases. This also represents the loss of cooperativity because a noncooperative system does not depend on a critical concentration.^27^ The change in *c*_c_ does, hence, not mean that higher concentrations of αS are required for multimers to form; instead, it marks a transition from a cooperative to a noncooperative multimer self-assembly process. The loss of cooperativity with increasing 14-3-3τ concentration shows that the interaction with 14-3-3τ interferes with the αS multimerization process, opening up a pathway toward the formation of different multimer species, containing both αS and 14-3-3τ (Figure 4D). These mixed-protein multimers are not on the pathway to amyloid formation.

### αS Multimers in the Presence of 14-3-3τ

To compare the self-assembled multimeric αS species that are found in the presence and absence of 14-3-3τ, we performed SM burst detection experiments on labeled αS in the presence of 14-3-3τ. As in the experiments in the absence of 14-3-3τ, shown in Figure 3C, we determined the passage times (*t*_passage_) through the optical volume for individual monomers and multimers in the presence of 14-3-3τ (Figure 5B). As before, we used picomolar concentrations of αS488 and measured the fluorescence passage times in a total of 110 μM αS, but now in the presence of 10 μM 14-3-3τ (Figure 5B). From the data, we again determine Δ*P*(*t*_passage_), the change in the distribution of passage times of multimers compared to monomers but now in the presence of 14-3-3τ. The addition of 14-3-3τ does not prevent αS multimerization; Δ*P*(*t*_passage_) also shows a shift from short to long passage times. The longer passage times are now, however, dominated by passage times close to 600 μs. In fact, calculating the average passage time for both the negative and positive part of Δ*P*(*t*_passage_) shows that the average passage time <600 μs remains constant (monomer contribution), while the average passage time >600 μs (multimer contribution) decreases in the presence of 14-3-3τ. Considering that 14-3-3τ does not bind αS monomers in this concentration range and that binding of the relatively large 14-3-3τ protein should increase the passage time, we conclude that the αS multimers formed in the presence of 14-3-3τ are smaller than the ones formed in absence of 14-3-3τ.

**Figure 6:**
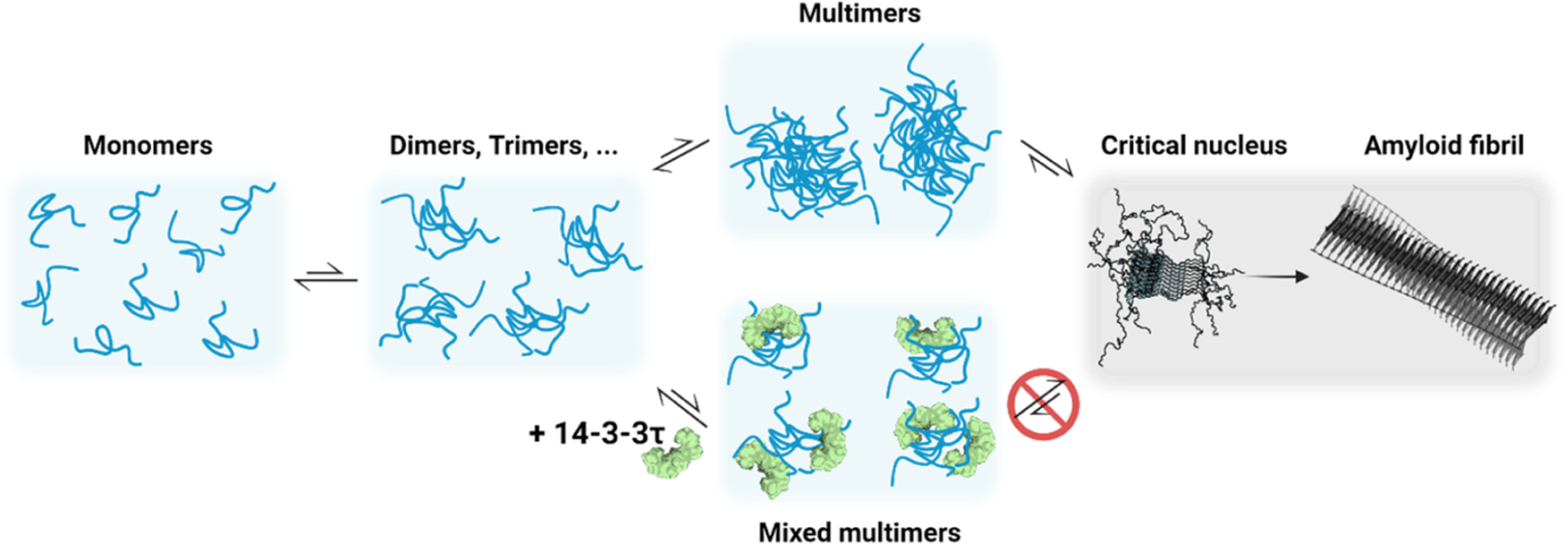
Cartoon depicting the proposed mechanism of multimerization of αS in the presence and absence of 14-3-3τ. In the presence of 14-3-3τ, mixed multimers of αS and 14-3-3τ are formed, which compete with pure αS multimers. The mixed multimers cannot develop into a critical nucleus for fibril formation. The formation of mixed αS/14-3-3τ multimers thereby deflects the aggregation of αS into amyloid fibrils.

Not only did the passage times of the multimers change, but we also observed a change in the fraction of αS present in monomers and multimers. In the presence of 14-3-3τ, a larger fraction of the fast diffusing species (passage times <600 μs) was converted into slower diffusing species (passage times >600 μs). In the absence of 14-3-3τ, 5% of the αS monomers was present in the slowly diffusing species, this increased to 8% in the presence of 14-3-3τ. We, therefore, conclude that in the presence of 14-3-3τ, not only smaller but also more multimers are formed (Figure 6). The smaller size of the αS multimers may not be purely the result of a lower aggregation number; binding of 14-3-3τ may also result in multimer compaction. For αS_30_, we indeed observe an indication of compaction in the presence of 14-3-3τ in bulk FRET experiments (SI, Figure S9).

## Discussion

We investigated the initial stages of αS multimerization, exploring the mechanisms that ultimately lead to the formation of αS amyloid fibrils. Utilizing MST and SM burst analyses, we observed that the initial αS multimerization is a nucleation-dependent process. We find that αS cooperatively assembles into dynamic multimers at a critical concentration. These multimers are metastable and precede the formation of an aggregation-prone nucleus for amyloid fibril formation (Figure 6).

The early αS multimers share properties with condensates observed in liquid–liquid phase separation.^41,42^ They are dynamic and liquid-like, and the αS proteins within them likely do not maintain fixed, well-defined conformations. Our findings suggest that transient multivalent interactions between αS molecules facilitate the cooperative assembly into multimers, classifying them as nanoscale condensates. Over time, these condensates may transform into nuclei for fibril formation through either structural rearrangements or growth.

Our results align with the Jovin group’s observations that amyloid aggregation initiates in colloidal aggregates of αS.^43^ They complement recent findings on the formation of nanoscale αS condensates evolving into larger structures and support the hypothesis that amyloid fibril aggregation is preceded by a condensate phase.^44,51^ Interestingly, the condensates that we observed are nanoscopic and spontaneously appear in solution even in the absence of crowding agents. The nanoscopic αS condensates potentially represent smaller analogs of the microscopic condensates commonly studied in this field.^45^

The addition of 14-3-3τ significantly alters the formation of early nanoscale αS condensates. Specifically, the presence of 14-3-3τ leads to the formation of mixed condensates of αS and 14-3-3τ. These co-condensates are formed via a noncooperative assembly process. This change from a cooperative to a noncooperative condensation mechanism is accompanied by the formation of more numerous but smaller co-condensates. We hypothesize that the co-condensation of αS with 14-3-3τ is driven by multivalent interactions. This agrees with what is reported for co-condensation of Tau with 14-3-3ζ.^46^ This co-condensation is also consistent with the idea that 14-3-3 proteins are potential regulators of liquid–liquid phase separation.^11,47^

Together, our findings indicate that the formation of αS/14-3-3τ co-condensates competes with the formation of pure αS condensates. Crucially, this competition deflects αS from the amyloid formation pathway. The co-condensates are not on the pathway to the formation of a critical nucleus for fibril formation, and therefore, co-condensation delays the formation of disease-associated amyloid structures. We show a mechanism where the loss of cooperativity in αS co-condensation allows for outcompeting the formation of pure αS condensates that can develop into aggregation-prone nuclei for amyloid fibril formation (Figure 6).

Our study, distinct from prior research, emphasizes the significance of interactions between chaperone proteins and aggregation-prone proteins within condensates at the nanoscale. It seems that nature passively inhibits amyloid formation at this initial stage, not requiring active processes that consume ATP. This finding is consistent with earlier studies on tau aggregation with 14-3-3ζ^48^ and both huntingtin and amyloid-β aggregation with DNAJB6,^42,49^ focusing on microscopic condensates. Our observations, together with these studies, indicate that chaperones might commonly use co-condensate formation as a strategy to hinder disease-associated protein aggregation.

In conclusion, our findings provide new insights into a primary defense mechanism against amyloid formation. This understanding could pave the way for novel approaches in disease detection and prevention, like modulating protein–protein interactions,^50^ and offers a new perspective on molecular mechanisms that control protein aggregation. Such molecular mechanisms could satisfy the urgent need for novel molecular concepts for pharmacological intervention in PD and related neurodegenerative diseases.

## Supporting Information Available

αS aggregation curves; thermal shift assay; SM burst analysis and passage times; confidence intervals for MST fits; FCS on αS_30_; negative controls multimerization; and FRET on αS_30_ in the absence and presence of 14-3-3τ (PDF)
