# Supplementary material for: 14-3-3τ as a Modulator of Early α-Synuclein Multimerization and Amyloid Formation
Source: ACS Chem Neurosci. 2024 Apr 18;15(9):1926–36. doi: 10.1021/acschemneuro.4c00100 (PMC11066837; doi:10.1021/acschemneuro.4c00100)
Supplement: Supplementary file 1 — cn4c00100_si_001.pdf [file cn4c00100_si_001.pdf]

Supplementary Information

Title: 14-3-3τ as a modulator of early α-synuclein multimerization and amyloid formation.

Authors: Gobert Heesink[1], Maxime C.M. van den Oetelaar[2], Slav A. Semerdzhiev[1], Christian Ottmann[2], Luc Brunsveld[2], Christian Blum[1], Mireille M.A.E. Claessens[1]

1: Nanobiophysics, Faculty of Science and Technology, MESA + Institute for Nanotechnology and Technical Medical Centre, University of Twente, Enschede 7500 AE, The Netherlands

2: Institute for Complex Molecular Systems, Eindhoven University of Technology, Eindhoven 5600 MB, The Netherlands

*Corresponding authors: c.blum@utwente.nl, m.m.a.e.claessens@utwente.nl

ThT aggregation assay

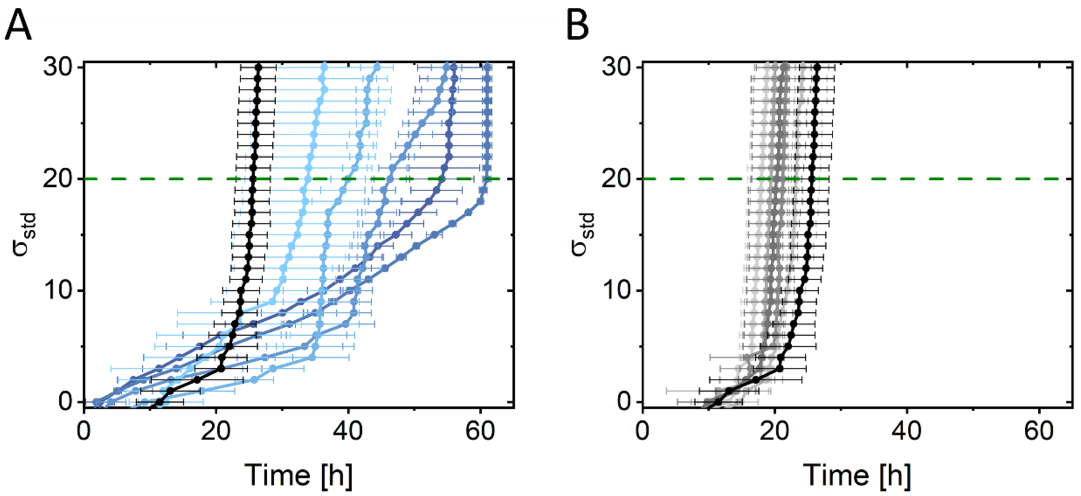

Figure S1. αS aggregation curves in absence (grey) and presence (blue) of 14-3-3τ. The thioflavin-T intensity is plotted relative to the standard deviation of the background signal (σ_{std}) as function of time. The threshold to determine the lag time is given at σ_{std} = 20, plotted in green. Each condition represents the time average of a triplicate and the error bars represent their standard deviation. **A**. The black curve corresponds to the aggregation of 100 µM αS. The blue curves correspond to the aggregation of 100 µM αS in presence of 5 µM (light blue), 10 µM, 15 µM, 20 µM and 25 µM (increasingly darker blue) 14-3-3τ. The presence of 14-3-3τ delays the aggregation lag time, with higher concentrations of 14-3-3τ resulting in a stronger delay. **B.** The αS aggregation curves corresponding to the aggregation of 100 µM (black), 95 µM, 90 µM, 85 µM, 80 µM and 75 µM (light grey) total αS. The aggregation lag time does not change significantly between αS concentrations of 75 µM and 100 µM.

Thermal shift assay

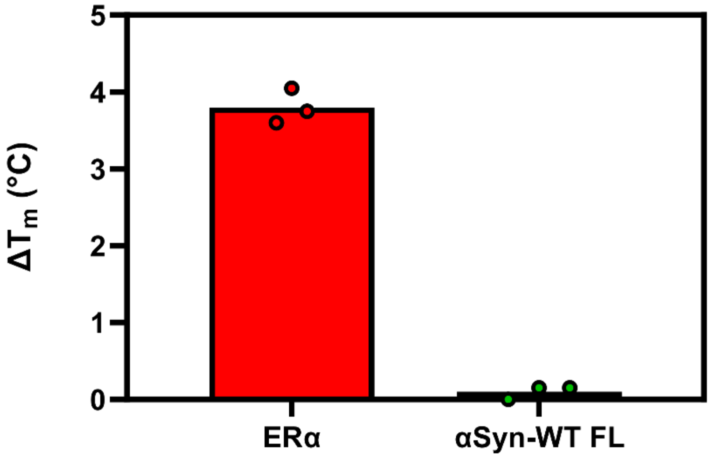

Figure S2. Change in melting temperature (ΔT_m) determined in a thermal shift assay of 14-3-3τ in presence of the known 14-3-3 binding partner Estrogen receptor alpha (ERα) or full-length αSyn-WT, relative to 14-3-3τ alone (53 °C). We observe an increased melting temperature (T_m) in presence of ERα, indicating interaction (positive control), while we observe no change in T_m in presence of αSyn-WT, indicating no interaction. Experiments are performed in duplicate three times. ΔT_m is calculated per experiment.

Single molecule burst analysis

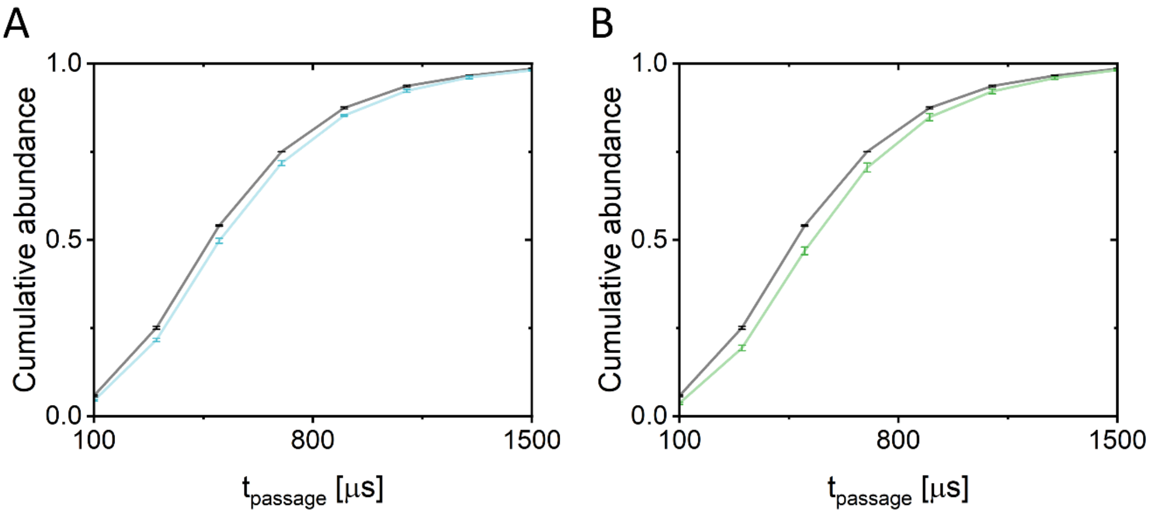

Figure S3. A. Normalized cumulative histograms of the passage times at low total αS concentration (grey) and high total αS concentration in absence of 14-3-3τ (blue), or **B.** presence of 10 µM 14-3-3τ (green). Error bars represent the standard deviation from three different measurements.

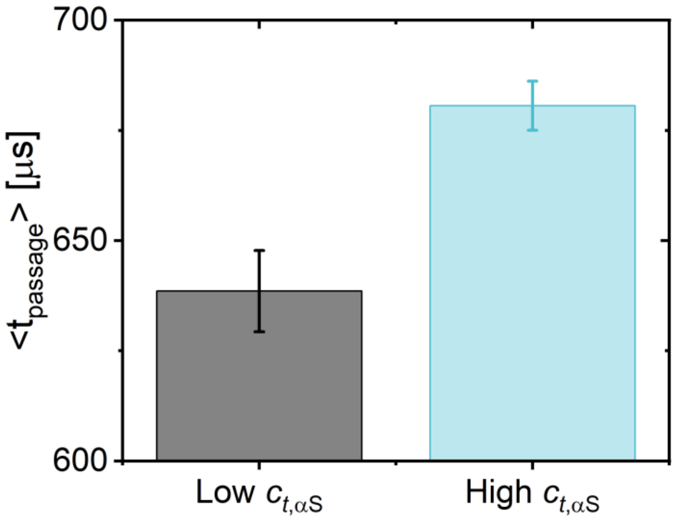

Figure S4. Average passage times at low total αS concentration (grey) and high total αS concentration (blue), corresponding to the data in Figure S3A. We find 639 ± 9 µs and 681 ± 6 µs for low and high total αS concentration, respectively. The increase in $\langle t_{passage} \rangle$ shows an increased population of slower diffusing, and hence larger species at high αS concentration. Error bars represent the standard deviation from three different measurements.

Robustness of MST experiments and model fits.

To study the robustness of the results derived from MST experiments on the multimerization of αS, we prepared and measured three independent samples of 50 nM αS-AF488 in presence of increasing amounts of αS-WT, performed at 37 °C. The MST results were fitted to the self-assembly model and parameters derived from the fits are presented in Figure S5.

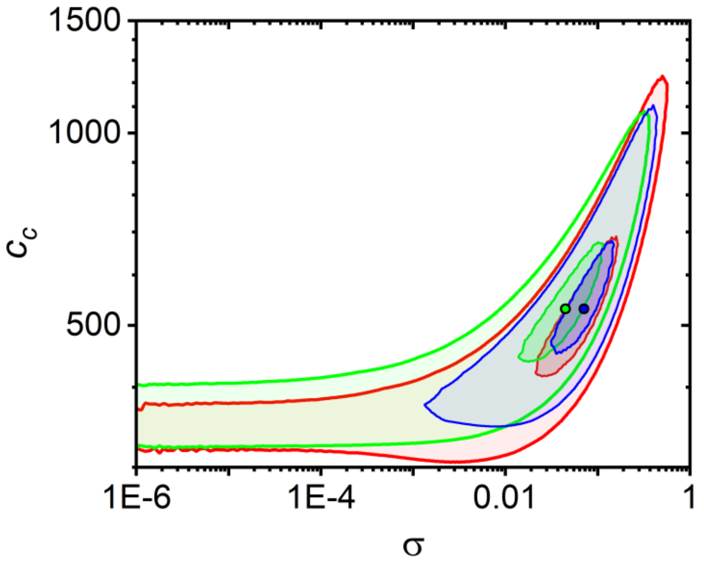

Figure S5. The self-assembly model fit result of three independently prepared and measured samples of 50 nM αS-AF488 in presence of increasing amounts of αS-WT up to 110 µM, performed at 37 °C. The coloured dots indicate the fitted values: (σ_1, $c_{c,1}$) = (0.07, 531 nM), (σ_2, $c_{c,2}$) = (0.04, 531 nM) and (σ_3, $c_{c,3}$) = (0.07, 531 nM). The dark and light shaded areas in corresponding colours show the 0.5th and 3rd percentile confidence intervals, respectively. Both the fitted values and shape of the confidence intervals agree very well between the triplicates.

Additionally, we studied the influence of each measured individual data point in a single measurement series on the fitted MST multimerization data. We therefore performed 5 repeats of the MST measurements on one sample containing 50 nM αS-AF488 and increasing amounts of αS-WT at 23 °C. We fitted the resulting average MST multimerization curve (Figure S6A) and the individual MST multimerization curves (Figure S6B-F) to the self-assembly model. The shaded areas, representing the 0.5th and 3rd percentile confidence intervals of the fits, are similarly shaped independent of the exact values obtained for σ and c_c.

Next we shuffled the data sets and created 500 additional MST multimerization curves from the 5 repeats by randomly selecting data points for each αS-WT concentration. These data sets were again fitted to the self-assembly model. Indeed, the resulting values of σ and c_c follow the confidence interval areas (Figure S6) as expected.

Together, the data presented in Figure S5 and Figure S6 evidences the robustness of the experiments and data analysis. Note that the shape of the confidence intervals may represent the characteristics of the multimerization curve even more accurately than the exact fitted values. A change in the shape of the confidence intervals, like we observe in presence of increasing amount of 14-3-3τ (Figure 5A), therefore also shows the different multimerization characteristics.

Finally, we find that the standard deviation of the measured MST response at given αS-WT following from the 5 repeats is smaller than the standard deviation determined based on the intra-variability for an individual measurement (see Methods section). Hence, the intra-variability serves as a fair, fast and easy approximation of the actual variance of the measurement, or may even overestimate it.

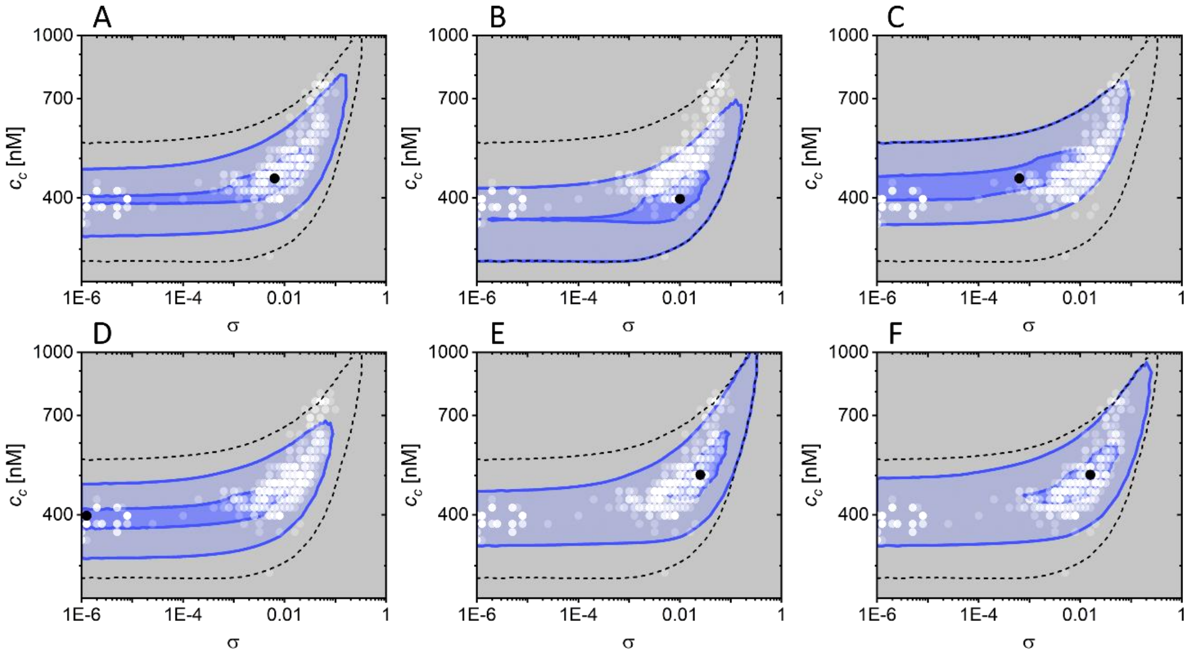

Figure S6. A. The self-assembly model fit result of 50 nM αS-AF488 + (3 nM – 110 µM) αS-WT performed at 23 °C. The dark and light blue shaded areas indicate the 0.5th and 3rd percentile confidence intervals, respectively. The fit is performed on the average MST response of a 5 times repeated measurement at 60% IR laser power. The white dots represent fit results performed on 500 MST multimerization curves with data randomly selected from the 5 repeats. Brighter dots indicate multiple occurrences. **B-F.** Results of fits to the 5 individual repeats. The black dashed line indicates the minimum and maximum critical concentration at given σ based on the 3rd percentile confidence intervals of **B-F**.

Fluorescence correlation spectroscopy (FCS) experiments on αS$_{30}$

To obtain a rough indication for the aggregation number of the αS multimers formed in the SM and MST experiments, we determined the diffusion passage time of αS$_{30}$, a higher order αS species of known aggregation number. We labelled αS$_{30}$ with AF488 as described for the labelling of αS$_{30}$-488/568 (see Materials and Methods), but with approximately 1 αS-AF488 per 3 αS$_{30}$. FCS measurements were performed on the same setup as the single molecule burst detection experiments (see Materials and Methods). Fluorescence fluctuation traces were measured for 180 s. We calculated the fluorescence autocorrelation curve for lag times between 0.03 ms and 1000 ms. We fitted this curve with a pure diffusion model and determine a diffusion time of 900 µs for αS$_{30}$. This analysis was performed with the SymphoTime64 software (PicoQuant).

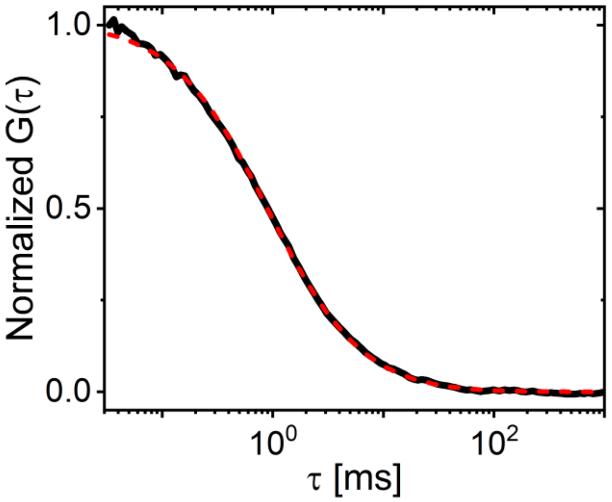

Figure S7. FCS autocorrelation curve for αS$_{30}$. The normalized G(τ) is plotted as function of the correlation lag time (τ). The curve is normalized to G(τ = 0.03 ms). The curve was fitted to a single species diffusion model with a diffusion time of 900 µs (red).

Negative control effect on αS multimerization

We used PEG20k as a negative control. PEG20k does not interact with αS and should therefore not affect the multimerization at the micromolar concentrations typically used for interaction studies.

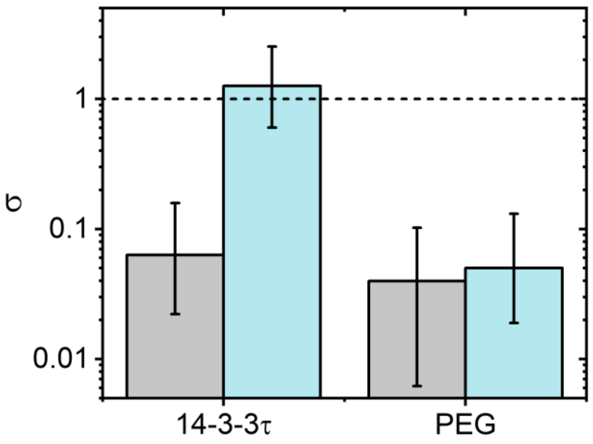

Figure S8. Value of σ for αS multimerization in absence (grey) and presence (blue) of either 14-3-3τ or PEG20k at comparable concentrations. The dashed line indicates the transition between cooperative and non-cooperative multimerization. Presence of 14-3-3τ results in non-cooperative multimerization of αS. A comparable concentration of PEG20k does not change αS multimerization. The error bars indicate the 0.5^{th} confidence interval for the fitted σ.

14-3-3τ may compact αS multimers

To study possible conformational changes in αS multimers upon binding 14-3-3τ, we measured the FRET emission of a double labelled αS FRET probe, αS-488/568, incorporated in an αS oligomer of approximately 30 monomers (αS_{30}). Emission spectra of αS_{30}-488/568 were recorded with a spectrophotometer (Fluoromax 4, Horiba Jobin Yvon – Edison, NJ USA) at 100 nM donor concentration in 10 mM Tris (pH 7.4) and 10 mM NaCl. The total concentration of αS_{30} was 3.3 µM (monomer equivalent). αS_{30}-488/568 was re-measured after adding 14-3-3τ to a final concentration of 3 µM αS_{30}-488/568 (monomer equivalent) and 2 µM 14-3-3τ, and 5 minutes incubation. High performance quartz glass cuvettes (105-251-15-40, Helma Analytics) were saturated with 5 µM αS-WT and rinsed twice with buffer before measurement. Samples were excited at 460 nm and emission was detected between 475 nm and 700 nm with an increment of 1 nm and integration time of 0.1 s. Excitation and emission slits were set to 5 and 3 nm, respectively. Presence of acceptor was validated by excitation at 550 nm and emission detection between 565 and 700 nm, with identical increment, integration time and slit sizes.

We find an increased FRET signal when adding 14-3-3τ to αS_{30}-488/568 (Figure S9). This shows binding of 14-3-3τ to αS_{30}-488/568 and indicates compaction of αS_{30} upon binding with 14-3-3τ. Thus, 14-3-3τ may compact αS multimers upon binding.

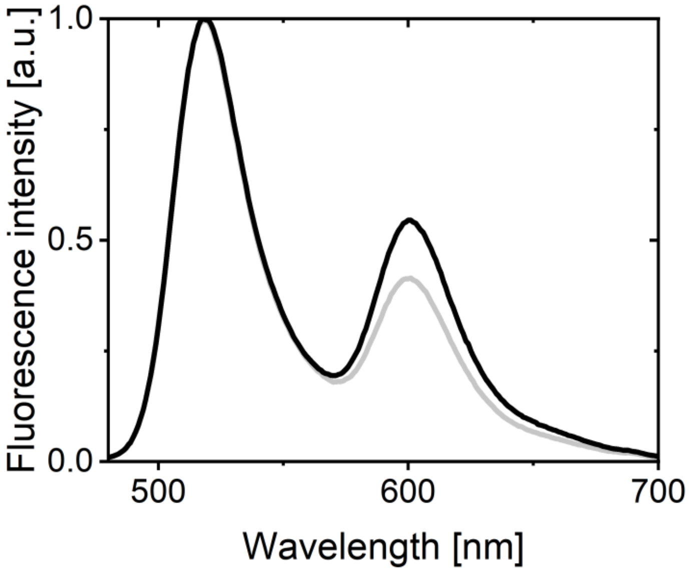

Figure S9. Normalized fluorescence emission of αS_{30}-488/568 in absence (grey) and presence of 2 µM 14-3-3τ (black). The sample was excited at 460 nm and the emission is normalized to the maximum fluorescence intensity peak at 517 nm. Addition of 14-3-3τ results in a higher FRET signal, indicating compaction of αS_{30} upon binding with 14-3-3τ.
